# Return to Sport After Anterior Cruciate Ligament Reconstruction Among Physically Active Adults

**DOI:** 10.7759/cureus.39850

**Published:** 2023-06-01

**Authors:** Abdulrahman J Korkoman, Bader Aljadaan, Anas Alqarni, Abdullah A Alshomrany, Abdullah N Almuawi, Abdullah F Alhalafi, Abdulmohsen N Alshahrani, Masoud M Alqahtani, Kady Althunayan

**Affiliations:** 1 Department of Orthopedic Surgery, University of Bisha, Bisha, SAU; 2 Department of Orthopedic Surgery, Prince Sultan Military Medical City, Riyadh, SAU; 3 College of Medicine, University of Bisha, Bisha, SAU

**Keywords:** arthroscopic acl reconstruction, anterior cruciate ligament (acl) injuries, anterior cruciate ligament (acl), anterior cruciate ligament (acl) reconstruction, return to sport

## Abstract

An anterior cruciate ligament (ACL) is one of athletes' most severe and frequent knee ligament injuries. The primary function of the ACL is preventing excessive anterior tibial translation, and it limits varus/valgus stress when the knee is in full extension and rotatory movements. Returning to sport after an ACL injury is a crucial aim of ACL reconstruction (ACLR). Multiple factors, modifiable and nonmodifiable, can influence the time to return to sport. This study aimed to discuss factors that affect optimal return-to-play (RTP) timing, symptom recurrence, and long-term consequences of an ACL injury.

This is a cross-sectional study involving patients who are following in orthopedic surgery outpatient clinics with a history of ACLR at least six months before surgery and not beyond six years after surgery. Participants received a survey about their sociodemographic data, details of the type and site of injury, and ACL return to sport before and after reconstruction scale. Full data description and testing of dependent variables against participant variables using two-sided tests were performed with a significance level of *P* ≤ 0.05.

The study involved 129 participants, of which the majority were male Bisha residents aged 20 to 29 years. The study found that the right leg was the most commonly injured, with the dominant leg being the most frequently reconstructed due to problems with knee function. Before the injury, most participants ran, cut (quick changes of direction during running), decelerated, and pivoted activities four or more times per month. However, physical activities notably reduced after ACLR. Age and body mass index (BMI) showed statistical significance related to the likelihood of returning to physical activities.

The study found a significant reduction in the frequency of activities such as cutting, deceleration, and running after ACLR. Age was identified as a predictor affecting the likelihood of returning to the sport, with older patients being less likely to return than younger ones.

## Introduction

The rotational and anteromedial stability of the knee joint is maintained by the anterior cruciate ligament (ACL) and posterior cruciate ligament (PCL) [1]. ACL is one of the most frequent and serious knee ligament injuries that sports medical professionals evaluate. They are more common in young, active people who play sports that require jumping, pivoting, and direction-changing, such as football, basketball, handball, and volleyball. In the United States, it has been estimated that 250,000 ACL tears take place every year [2]. Cruciate and collateral ligaments injury are common in Saudi Arabia, with a prevalence of about 26.2%, with ACL injury being the most common [3]. Women participating in athletics are two to eight times more likely to sustain ACL injury than their male counterparts in the same landing and pivoting sports [2,4]. The primary function of ACL is preventing excessive anterior tibial translation, particularly by the anteromedial bundle. Second, it limits varus/valgus stress when the knee is in full extension and rotatory movements are controlled primarily by the posterolateral bundle [5].

Multiple factors classified as modifiable and nonmodifiable are thought to influence ACL injuries. Some of the most common modifiable factors are neuromuscular control and landing biomechanics [6,7]. The risk of injury and recurrence increases if these factors are not addressed. Acute management after ACL injury consists of ligament reconstruction predominantly in young, high-level athletes participating in high-demand sports and those with persistent functional knee instability [8,9]. Nonsurgical treatment can be an option for special populations, such as athletes who participate in sports that do not involve a change of direction(e.g., golf) and those without persistent or significant instability. A comprehensive rehabilitation program is an essential component of ACL injury management, although not established in many studies [10]. Several factors must be evaluated when determining whether the patient should return to play (RTP) after injury or ACL reconstruction (ACLR). Recently published literature has established that RTP could be slower than was previously reported and that better results are obtained nine months after reconstruction [11,12]. The average time to resume activity is six to 12 months after ACLR surgery; however, the nature of the postinjury activity and the patient’s rehabilitation progress may affect the average time to return to activity [13]. According to Ardern et al., 90% of patients had a successful outcome after ACLR surgery [14]. Irrespective of the high rate of successful surgical outcomes and physical improvement with rehabilitation, only 63% of patients returned to their pre-injury activity level [15,16]. This study aims to discuss factors that affect optimal RTP timing, symptom recurrence, and long-term consequences of an ACL injury. The most important question an athlete will probably ask after an ACL injury is as follows: When can I play again? The answer to this issue is frequently not straightforward and ought to be based on many objective standards. These include the patient's clinical presentation, isokinetic and functional testing, timing and type of surgery, and data from verified surveys that evaluate subjective elements such as patient satisfaction and psychological preparation for a return to sports.

## Materials and methods

This is a descriptive cross-sectional study involving patients following up in the orthopedic surgery outpatient clinic at King Abdullah Hospital, Bisha, Saudi Arabia, after their ACL reconstruction operation, from January 1, 2022, to January 1, 2023. The study was conducted in Bisha Province, Southwest Saudi Arabia. Patients who recently underwent primary ACLR or had the surgery at least six months earlier and had completed rehabilitation and had an interval of not more than six years after surgery were included. Patients with multiple, bilateral knee injuries, aged <18 years, or a history of a low back injury (as it might affect rehabilitation) were excluded.

A structured self-administered questionnaire was distributed among all patients who matched the study's inclusion criteria after obtaining their names and file numbers from the registry database of King Abdullah Hospital. 

All participants underwent surgery as clinically indicated with a quadrupled hamstring graft. The questionnaire contained sociodemographic data, details of the type and site of injury, dominant leg, reason for reconstruction, history of ACLR, time of injury that occurred in months, time since ACLR in months, and return to sport before and after ACLR scale.

IBM Statistical Software for Social Sciences (SPSS) for Windows, Version 25 (IBM Corp., Armonk, NY, USA) was used to conduct statistical analyses. We analyzed the data as follows: First, we gave a full description of the dataset of numbers, frequencies, and percentages. Second, we tested the dependent variables separately against each of the participant predictor variables through the use of either cross-tab procedure. All tests were two-sided, and a *P*-value ≤0.05 was considered to be statistically significant.

## Results

A total of 129 patients participated in this study. Of the 129 participants, 126 (97.67%) were males and three (2.33%) were females. All the participants in this study were Bisha residents. The majority of the participants were aged between 20 and 29 years (55.81%), while the least represented age group was those above 40 years (4.65%). Other important variables like weight and height were obtained, and the corresponding body mass index (BMI) was computed. The weights of the respondents were between 50 and 100 kg, with the majority of respondents having 70 to 79 kg (51.16%), while the least proportion was those above 90 kg (6.98%). Regarding the BMI, about half of the respondents had an average BMI, while 26.36% were overweight and 14.73% were obese (Table 1).

**Table 1:** Sociodemographic characteristics of respondents.

Variable	Category	Count (*n*)	Percentage (%)
Sex	Male	126	97.67
	Female	3	2.33
Ethnicity	Saudi	129	100
	Non-Saudi	0	0
Age group (years)	<20	15	11.63
	20-29	72	55.81
	30-39	36	27.91
	40-49	6	4.65
Height (cm)	Below 160	3	2.33
	161-170	78	60.47
	171-180	48	37.21
Weight (kg)	50-59	12	9.30
	60-69	30	23.26
	70-79	66	51.16
	80-89	12	9.30
	90-100	9	6.98
Body mass index	Obese	19	14.73
	Overweight	34	26.36
	Normal weight	65	50.39
	Underweight	7	5.43

The right leg experienced the most cases (67.44%) compared to the left leg (32.56%). It was observed that the dominant leg is the most commonly injured (65.12%). When the questions on the reason for reconstruction were posed, the majority of the respondents (29.46%) responded to their choice of surgery as that there was a problem with knee functioning (standing and walking). The other reasons stated included pain (9.30%), instability (13.95%), weakness (4.65%), and desire to return to normalcy (25.58%). Most respondents' injuries had occurred within the last two years (72.09%), and the reconstruction was conducted within the same period (Table 2).

**Table 2:** Injury data of respondents. ACL, anterior cruciate ligament

ACL injury data		Count (*n*)	Percentage (%)
Injury site	Right	87	67.44
	Left	42	32.56
Injury at the dominant leg	Yes	84	65.12
	No	45	34.88
Reason for reconstruction	Pain	12	9.30
	Instability	18	13.95
	Weakness	6	4.65
	Problem with knee functioning	38	29.46
	Desire to return to normalcy	33	25.58
	Fear of complications	18	13.95
	Other Reasons	4	3.10
Time when the injury occurred	One year, one day to two years ago	93	72.09
	Two years, one day to four years ago	24	18.60
	Four years, one day to six years ago	3	2.33
	Seven years, one day ago or more	9	6.98
Time since the ACL reconstruction	One year, one day to two years ago	111	86.05
	Two years, one day to four years ago	12	9.30
	Four years, one day to six years ago	6	4.65

Participants' physical activities declined immensely after an ACL injury. Before the injury, most participants would run four or more times a month, which reduced from 18.60% to 9.30% (about half) after reconstruction. Additionally, none of the respondents (0.00%) could do cutting and pivoting after reconstruction (Table 3).

**Table 3:** Summary of activities before and after ACL surgery. ACL, anterior cruciate ligament

Activities	Duration	Before injury		After injury	
		n	%	n	%
Running	Less than once a month	36	27.90	54	41.90
	Once a month	21	16.30	39	30.20
	Once a week	18	14	12	9.30
	Two to three times a week	30	23.30	12	9.30
	Four or more times a week	24	18.60	12	9.30
Cutting	Less than once a month	54	41.90	78	60.50
	Once a month	36	27.90	33	25.60
	Once a week	27	20.90	15	11.60
	Two to three times a week	12	9.30	3	2.30
	Four or more times a week	0	0	0	0
Deceleration	Less than once a month	54	41.90	66	51.20
	Once a month	36	27.90	42	32.60
	Once a week	30	23.30	18	14
	Two to three times a week	6	4.70	3	2.30
	Four or more times a week	3	2.30	0	0
Pivoting	Less than once a month	54	41.90	87	67.40
	Once a month	36	27.90	30	23.30
	Once a week	24	18.60	9	7
	Two to three times a week	12	9.30	3	2.30
	Four or more times a week	3	2.30	0	0

There was a statistical significance between age and return to physical activity. Statistical significance was also observed in BMI. It indicates that these variables affect the likelihood (correlation) of returning to physical activities. The other variables did not show any statistical significance (Table 4).

**Table 4:** Relationship between demographic variables and ability to return to sport (physical activities). *Statistical association.

Variable	Category	Count (*n*)	Percentage (%)	P
Sex	Male	126	97.67	0.675
	Female	3	2.33	
Ethnicity	Saudi	129	100	0.557
	Non-Saudi	0	0	
Age group (years)	<20	15	11.63	0.001*
	20-29	72	55.81	
	30-39	36	27.91	
	40-49	6	4.65	
Height (cm)	<160	3	2.33	0.426
	161-170	78	60.47	
	171-180	48	37.21	
Weight (kg)	50-59	12	9.30	0.3992
	60-69	30	23.26	
	70-79	66	51.16	
	80-89	12	9.30	
	90-100	9	6.98	
Body mass index	Obese	19	14.73	0.001*
	Overweight	34	26.36	
	Normal weight	65	50.39	
	Underweight	7	5.43	

## Discussion

This study aimed to determine the prevalence of return to sport after ACLR among physically active adults in rural areas of Saudi Arabia. This study involved 129 respondents, with the vast majority of the participants being male (97.67%). ACL injuries were more common in the right leg (67.44%). This could be because the right leg is often the dominant leg, and it gets load asymmetry, increasing its susceptibility to ACL injury. Of the total number of respondents, 65.12% had ACL in the dominant leg. According to Ruedl et al., the dominant leg is a risk factor for ACL injury, and more than 68% of ACL incidences occurred in the dominant leg [17].

Based on this study, the main reasons athletes opted for the reconstruction were problems with knee function (29.46%) and the desire to return to normalcy (25.58%). There is consistency between our findings and a study conducted by Chantrelle et al., which claims that most ACL reconstruction cases are performed to stabilize the knee and the desire of athletes to return to normalcy [18]. However, Bousquet et al. ascertained that the main reason for ACLR is the prevention of joint degeneration and the strengthening of the leg muscle [19].

This study's results indicate that ACLR significantly reduced various activities like running, cutting, deceleration, and pivoting. After the reconstruction, the participants abridged the frequency of these activities. According to Pairot-de-Fontenay et al., running and pivoting significantly reduce after ACL injury but may resume normalcy over time [20]. Our results also indicate a statistical association between return to sport with variables such as age (*P *= 0.001) and BMI (*P* = 0.001). However, other demographic variables such as ethnicity did not predict the return to sport. These findings are consistent with a study conducted by Howard et al. in the United States, which asserted that younger athletes had a higher probability of returning to sport [21].

The duration after ACL is a crucial determinant in return to sport. Most athletes do not have a regular season after the injury. Great care should be considered after the injury, and athletes should be gradually introduced to build the intensity of their activities. The longer the duration after injury, the greater the chances of returning to normalcy [22]. The results from our study indicated that the vast majority of the respondents (72.09%) had the injury between one and two years ago. This implies that most of them would experience better cutting, deceleration, pivoting, and running over time. According to Alswat et al., the average duration of return to sport after an ACL injury is six to 12 months. However, the return to normalcy is gradual and varies from individual to individual [23].

The athlete's physical state is a key determinant of a triumphant return to sport. Still, the emotional or cognitive reaction to ACLR is a key factor. A study conducted by Raizah et al. in Saudi Arabia stated that kinesiophobia significantly affected activities after reconstruction [24]. According to our research, there was a significant improvement in athletic activities, but kinesiophobia could be an essential founding variable. A previous systematic review found a significant proportion of patients with ACL injury had a great fear of reinjury; this was detrimental to their journey of full recovery after the ACL reconstruction [14].

This study has some limitations. The study used a self-administered questionnaire. This study had a limited sample size that lacks sufficient female representation, which may affect the reproducibility in some countries where females have a higher contribution to sport. There is only a handful of prior research on the return to sport after ACL reconstruction; hence, the literature review was limited to a few studies. A lack of cooperation was observed with some respondents. Rehabilitation and psychological status could also play a role in return to sport and should be assessed further.

## Conclusions

The optimal performance after ACLR remains elusive. However, the vast majority of patients with ACL injuries return to normalcy. Based on our study, there was a significant reduction in the frequency of activities like cutting, deceleration, and running after ACLR. Several predictors affect returning to sports. Age is of particular interest as older patients were less likely to return to sports than young ones. Future efforts should study the possible association between rehabilitation and the psychological status of patients to study the variables that most likely affect the ability to perform unrestricted physical activity.
